# Fascin induces melanoma tumorigenesis and stemness through regulating the Hippo pathway

**DOI:** 10.1186/s12964-018-0250-1

**Published:** 2018-07-03

**Authors:** Jiaxin Kang, Jian Wang, Zhuang Yao, Yuanzhao Hu, Shijie Ma, Qin Fan, Feng Gao, Yan Sun, Jianwei Sun

**Affiliations:** 1 Guangdong Provincial Key Laboratory of Protein Function and Regulation in Agricultural Organisms, College of Life Sciences, South China Agricultural University, Guangzhou, 510642 China; 2 Tianjin Medical University Cancer Institute and Hospital National Clinical Research Center for Cancer, Key Laboratory of Cancer Prevention and Therapy, Tianjin’s Clinical Research Center for Cancer, Tianjin, China; 3 Institute of Agriculture and Life Sciences, Gyeongsang National University, Jinju, 52828 Korea; 4 Department of Cellular and Molecular Physiology, the Pennsylvania State University College of Medicine, Hershey, PA USA

**Keywords:** Fascin, Melanoma, Hippo, MST2, TAZ

## Abstract

**Background:**

Fascin is a F-actin bundling protein and its overexpression is correlated with poor prognosis and increases metastatic potential in a number of cancers. But underlying function and mechanism of fascin on tumorigenesis in melanoma remain elusive.

**Methods:**

The melanoma cell lines WM793 and WM39 were employed for the soft agar and sphere formation assay. Quantitative RT-PCR and Western blot were performed for identifying the gene expression at mRNA and protein levels, respectively. Co-IP and in vitro GST pulldown experiments were used to test the interaction between fascin and MST2.

**Results:**

Fascin regulates tumorigenesis and cancer cell stemness in melanoma through inhibition of the Hippo pathway kinase MST2 and the activation of transcription factor TAZ. Our data showed that fascin interacts with the kinase domain of MST2 to inhibit its homodimer formation and kinase activity. Depletion of fascin led to increase of p-LATS level and decrease of TAZ, but not YAP. We also demonstrated that fascin regulates melanoma tumorigenesis independent of its actin-bundling activity.

**Conclusions:**

Fascin is a new regulator of the MST2-LATS-TAZ pathway and plays a critical role in melanoma tumorigenesis. Inhibition of fascin reduces melanoma tumorigenesis and stemness, and thus fascin could be a potential therapeutic target for this malignancy.

**Electronic supplementary material:**

The online version of this article (10.1186/s12964-018-0250-1) contains supplementary material, which is available to authorized users.

## Background

Fascin, an actin-bundling protein, is a key element in tumor metastasis [1, 2] and has been shown to regulate assembly of actin bundles. Fascin bundles actin through three binding sites and generates protrusive force to drive cancer metastasis [3–5]. It is generally thought that the causal role of fascin in tumor metastasis predominantly depends on its actin-bundling activity. A number of recent studies demonstrated that fascin is a key regulator of mammary tumorigenesis and breast cancer cell stemness [6, 7]. However, its role in melanoma tumorigenesis and melanoma metastasis remains largely unknown.

Hippo pathway is an evolutionally conserved cascade and well-known function of this pathway is to control organ size. Recent evidence indicates that the Hippo pathway is also critical for tumorigenesis and cancer stem cell self-renewal [8, 9]. The core components of the Hippo pathway include serine/threonine kinase MST (Mammalian Sterile 20 Like kinase), LATS (large tumor suppressor kinase) and major downstream mediator YAP (Yes Associated Protein)/TAZ (Trascriptional Coactivator with PDZ-binding motif). Activation of two kinases, MST and LATS, leads to LATS-dependent phosphorylation of YAP/TAZ, limiting their stability, nuclear localization and transcriptional activity.

TAZ, is a WW domain-containing transcriptional co-activator that shares 50% sequence identity with YAP [10, 11]. In mammal, TAZ was reported to play more important role than YAP in cancer development. TAZ and YAP have also been reported to regulate cancer cell stemness and tumorigenesis in several human malignancies including lung and breast cancer and hepatocarcinoma [9, 12–14]. However, TAZ, but not YAP, was reported to play critical role in mesenchymal stem cell differentiation [15].

Melanoma is well known for its invasive and metastatic behavior. A recent study showed that TAZ expression level was higher than YAP in melanoma [16]. In addition, TAZ knockdown inhibits melanoma growth, suggesting that TAZ enacts a critical role in melanoma tumorigenesis and that TAZ could be a primary Hippo effector in melanoma.

It has been demonstrated that the Hippo pathway can be regulated by actin cytoskeleton [17]. Following actin polymerization, Hippo signaling is disrupted. In cancer cells, changes in actin cytoskeleton were shown to regulate YAP activity [18–20]. A recently crystal structure study revealed that MST2 phosphorylates LATS1 at its hydropholic motif T1079 site and that MST2 homodimer is required for MST2 kinase activity [21]. Here, we demonstrated that fascin interacts with MST2 and reduces MST2 homodimer formation and kinase activity, which leads to lower p-LATS and stabilizing TAZ. We also found that the regulation of TAZ by fascin is independent of fascin actin-bundling activity in melanoma. Our results uncover a new role of fascin i.e., fascin regulates TAZ stability through interacting with MST2 in melanoma.

## Methods

### Cell culture

Melanoma cell lines WM793 and WM39 were cultured in RPMI1640 medium supplemented with 10% fetal bovine serum (FBS) and penicillin/streptomycin. HEK293T cells were cultured in DMEM medium supplemented with 10% FBS and penicillin/streptomycin.

### Antibodies

The following antibodies were used in this study: fascin, (Santa Cruzsc-21,743); CD44 (CST 3570S); TAZ (CST 9261S); YAP (CST 9520); GFP (CST 2555S), phospho-LATS1-T1079 (CST,9101); Lats1 (CST 3147S), Lats2 (CST,5888S) and GAPDH (Sigma G8795).

### Soft agar assay

Cells (5 × 10^3^) were mixed with 1.0 ml of growth medium with 0.3% agarose and layered onto 1 ml of 0.5% agarbeds in 12-well plate. Cells were fed with 1 ml medium every 2 days for 4 weeks, and the colonies were stained with 0.02% iodonitrotetrazolium chloride (Sigma-Aldrich) and photographed. Colonies larger than 100 μm in diameter were counted as positive.

### Sphere formation assay

Cells were plated in ultralow attachment 96-well plate (Corning Inc.) at the density of 10,000 cells/ml (1000 cells/100 μl/well) in stem cell selective medium at 37 °C for 10 days. The spheres were observed using an automated Zeiss Observer Z.1 inverted microscope and images were acquired using the AxioCam MRm3 CCD camera and Axiovision version 4.7 (Carl Zeiss Inc., Germany). The number of spheres greater than or equal to 100 μm were counted. The sphere formation assay was performed twice in triplicate of each treatment.

### Plasmid construction and transfection

Fascin targeting sequence GAAGAAGCAGATCTGGA was inserted to lenti-CRISPR V2 vector according to previous report [22]. Wild-type fascin expression plasmid was generated by inserting full-length fascin cDNA into pLncx2 retrovirus vector at BglII and EcoRI sites. Fascin-S39E mutant was prepared using Quick Change Mutagenesis kit. Plpcx-flag-MST2 was generated by inserting Flag-MST2 cDNA into plpcx vector at XhoI and EcoRI sites. Full length and truncated fragments (1-313AA and 314-491AA) of MST2 were inserted to PET28a vector at BamHI and XhoI sites.

Plasmids of pEGFP-C3-MST2 (Plasmid #19056), pLenti-EF-FH-TAZ-ires-blast (Plasmid #52083) and pLenti-EF-FH-TAZS89A-ires-blast (Plasmid #52084) were obtained from Addgene.

### Western blot analysis

Total cell lysates were separated on 10%SDS-PAGE gel. After electro-transferring, membranes were blocked with non-fat dry milk for 30 min at room temperature. Following washing three times, the blots were incubated with primary antibodies and then peroxidase-linked anti-mouse IgG (Amersham Biosciences). The bands were detected by an ECL-plus Western blotting detection system (Amersham Biosciences)..

### qRT-PCR

Total RNA was extracted from cultured cells using QIAGEN RNaesy Mini kit and reverse transcription was performed using the ABI cDNA synthesis kit. Quantitative real-time PCR (qRT-PCR) was carried out with the Applied Biosystems 7900HT fast real-time PCR system using Applied Biosystems SYBR Green PCR master mix. Primers are as follows:

- TAZ q-PCR NS: 5′- AGCTCAGATCCTTTCCTCAATG-3′
- TAZ q-PCR CAS: 5′- TCCTGCGTTTTCTCCTGTATC-3’
- GAPDH q-PCR NS 5’-TGAAGGTCGGAGTCAACGG-3’
- GAPDH q-PCR CAS: 5’-AGAGTTAAAAGCAGCCCTGGTG-3’

All reactions were performed in triplicate, and each experiment was repeated three times.

### Co-immunoprecipitation (co-IP) assay

Following transfection of HEK293T cells with plpcx-FLAG-fascin and pEGFP-C3-MST2 for 36 h, the cells were lysed in a buffer [50 mM Tris (pH 8.0), 150 mM NaCl, 0.5% sodium deoxycholate, 1% Triton X-100,1 mM phenylmethylsulfonyl fluoride (PMSF)] and then incubated with M2 beads (anti-Flag antibody, Sigma) for 2 h at 4 °C. The beads were washed extensively in a buffer containing 50 mM Tris (pH 8.0), 150 mM NaCl, 1 mM PMSF and 1% NP-40 and the bound proteins were eluted and resolved on SDS-PAGE and detected with the antibodies indicated in the figure legend.

### GST pull-down assay

Purified GST or GST-fascin fusion proteins were immobilized on glutathione-sepharose beads and incubated with [^35^S]methionine-labeled MST2-His proteins at 4 °C for more than 2 h. The beads were then washed extensively and the bound proteins were eluted and separated on 10% SDS-PAGE and exposed to phospho-imager screen (Amersham) for autoradiography.

### Statistical analysis

Data were analyzed using GraphPad Prism 6.0 (GraphPad Software Inc., San Diego, CA, USA), and all results were expressed as means ± SEM. Statistically significant differences were determined using Student’s t-test for two-group analysis and one-way analysis of variance (ANOVA) for more than two groups. Statistical significance was defined as *P < 0.05, **P < 0.01 or ***P < 0.001.

## Results

### Fascin has an important role in melanoma tumorigenesis and melanoma stemness

**Fig. 1:**
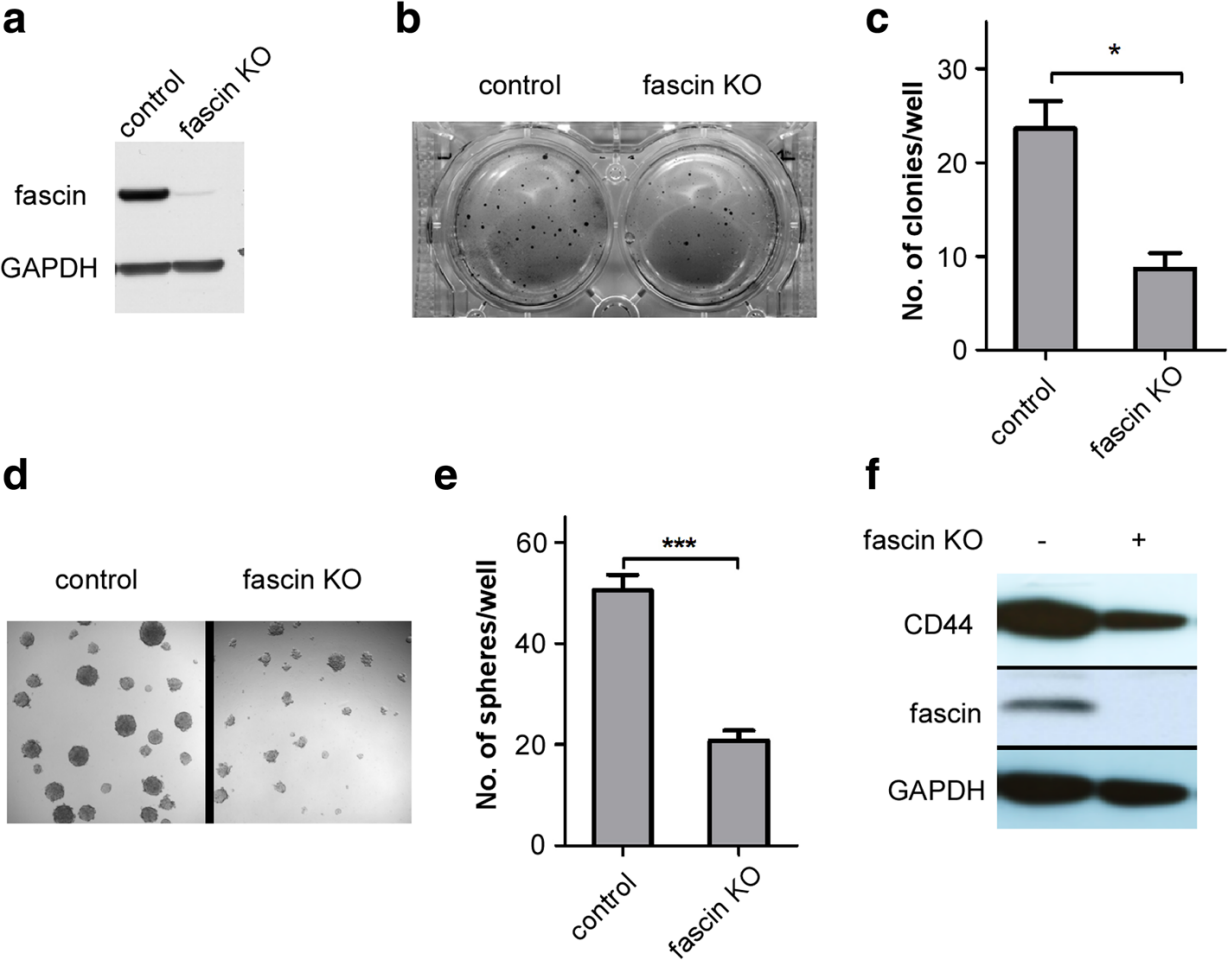
Fascin regulates melanoma tumorigenesis and stemness. **a** Western blot analysis of fascin in WM793 cells after treatment with fascin sgRNA and control sgRNA. **b** WM793 control and fascin KO cells were subjected to soft-agar colony-formation assay, and the colonies were stained with iodonitrotetrazolium chloride. **c** Quantification of colony numbers per well was shown in bar graph. Data represented as mean ± SD; *n* = 3 (*P < 0.05). **d** WM793 control and WM793 fascin KO cells were subjected to sphere formation assay. **e** Quantification of sphere numbers in WM793 control and WM793 fascin KO cells. Data represented as mean ± SD; *n* = 3 (***P < 0.001). **f** Western blot analysis of CD44 expression in control and fascin KO WM793 cells

Fascin has been shown to promote cancer progression in several human malignancies including breast and pancreatic carcinoma. However, its role in melanoma remains largely unknown. Thus we started to investigate the effect of fascin on melanoma tumorigenesis and melanoma stem cell growth. We first examined the expression level of fascin in a series of melanoma cell lines and found that fascin is highly expressed in the majority of melanoma lines (Additional file 1: Figure S1). We selected WM793 (a high fascin level cell line) and WM39 (a lower fascin level cell line) in our study. We knocked out fascin in WM793 cell line using CRISPR/CAS9 system after infection of the cells with lenti viruses of CRISPR/CAS9 fascin sgRNA and control CRISPR/CAS9 sgRNA. Western blot analysis showed that fascin was almost completely depleted in WM793 cells (Fig. 1a). To examine the role of fascin in melanoma tumorigenesis and melanoma sphere-forming capability, we performed soft agar colony formation and sphere formation assays. Our results showed that deletion of fascin in WM793 cells dramatically reduced anchorage-independent growth (Fig. 1b and c) and the number of melanoma sphere by more than 50% (Fig. 1d and e). To further confirm the role of fascin in regulation of melanoma stemness, we examined the expression level of CD44 in fascin knockout (KO) and control WM793 cells and found that CD44 level was significantly reduced in fascin KO cells (Fig. 1f). These results suggested fascin plays a pivotal role in melanoma tumorigenesis and melanoma stem cell growth.

### Fascin regulates melanoma tumorigenesis and stemness independent of its actin-bundling activity

**Fig. 2:**
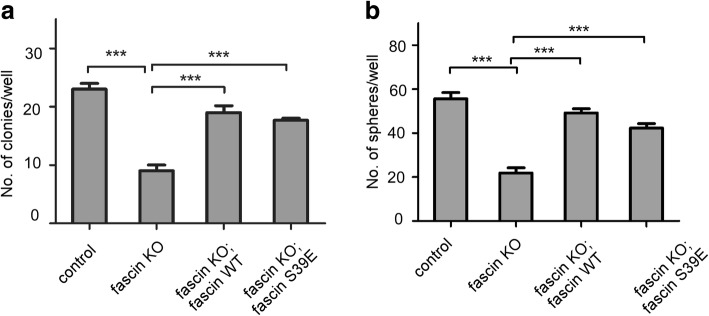
Fascin regulates melanoma tumorigenesis and stemness independent of its actin-bundling activity. **a** Soft agar assay was carried out in fascin-WT and fascin-S39E-reintrouced WM793 fascin KO cells. The number of colonies was counted in these cells. Data represent as mean ± SD; *n* = 3 (*P < 0.05, **P < 0.01). **b** Sphere formation assay was performed in fascin-WT and fascin-S39E-reintrouced WM793 fascin KO cells. The number of sphere was counted in these cells. Data represent as mean ± SD; *n* = 3 (***P < 0.001)

It has been well documented that a majority of fascin functions are mediated by its actin-bundling activity. Therefore, we examined if the effect of fascin on melanoma tumorigenesis and stemness is also dependent on its actin-bundling activity. We ectopically expressed fascin-wild type (WT) and loss actin-bundling activity mutant fascin-S39E in WM793 fascin KO cells. Soft agar and sphere formation assays revealed that both fascin-WT and fascin-S39E could fully rescue the effect of fascin knockout on anchorage independent growth (Fig. 2a) and sphere formation (Fig. 2b). Collectively, these results indicated that fascin regulates melanoma tumorigenesis as well as stemness independent of its actin-bundling activity.

### Fascin regulates the hippo pathway

**Fig. 3:**
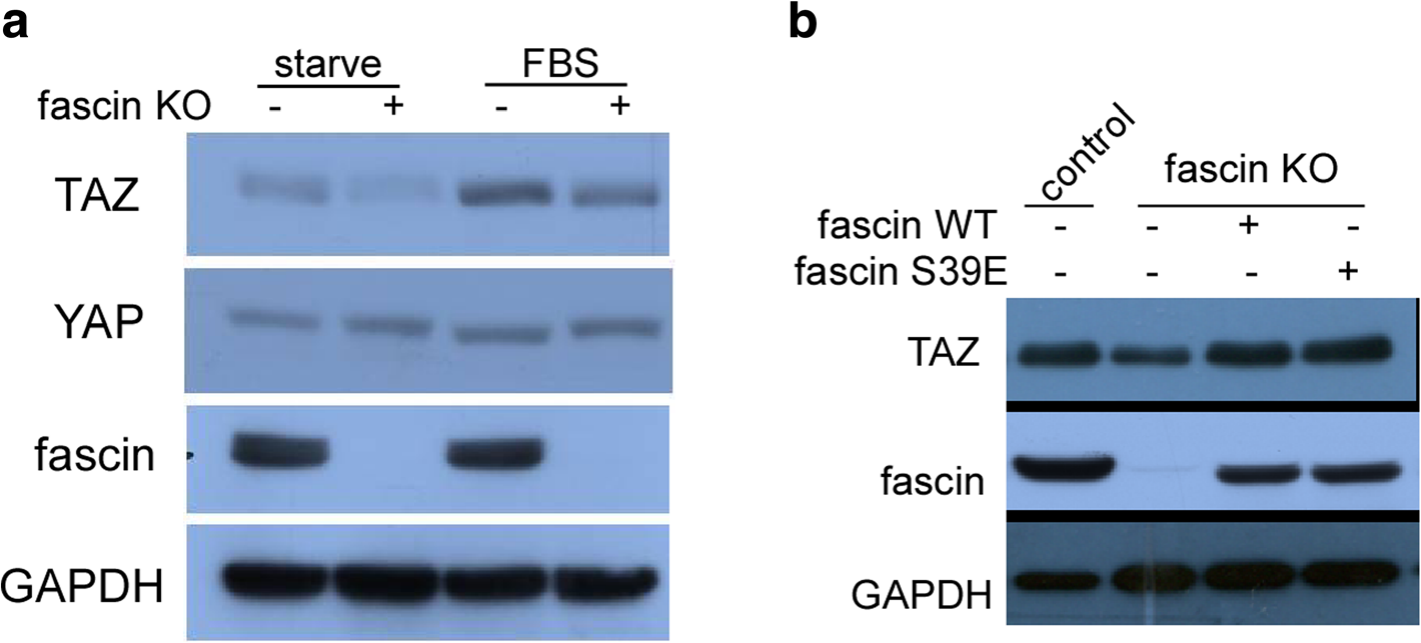
Fascin regulates TAZ protein level independent of its actin-bundling activity. **a** WM793 fascin KO cells were starved for 12 h and then cultured with/without FBS and then subjected to Western blot analysis with indicated antibodies. **b** WM793 fascin KO cells were reintroduced with fascin-WT and fascin-S39E and Western blot analysis was performed using indicated antibodies

While we and others have shown a critical role of fascin in melanoma and breast and pancreatic cancer [1, 23], the underlying molecular mechanism remains elusive. Because the Hippo pathway has been demonstrated as a key regulator of tumorigenesis and cancer cell stemness, we next scrutinized the possible connection between fascin and the Hippo cascade. Interestingly, we found that knockout of fascin (fascin KO) decreases TAZ protein expression level by more than 50% after serum starvation for 12 h. Even 2 h after adding back FBS to the medium, TAZ level in WM793 fascin KO cells was still much lower than that in control cells (Fig. 3a). However, Yes Associated Protein (YAP) level was not affected by fascin knockout, implying that fascin action in melanoma tumorigenesis and stemness could be mediated by TAZ but not YAP.

To further determine whether fascin regulates TAZ, we reintroduced fascin into WM793 fascin KO cells. Although fascin expression level in rescue cells was slightly lower than endogenous fascin level (Fig. 3b), ectopic expression of fascin was able to fully restores the TAZ level, further supporting the notion that fascin regulates TAZ in melanoma. We next investigated if fascin regulation of TAZ depends on its actin-bundling activity. We also introduced fascin-S39E into WM793 fascin KO cells and then assessed its effect on TAZ expression. Interestingly, we found that fascin-S39E, similar to wild-type fascin completely rescues TAZ expression in WM793 fascin KO cells. These results indicated that fascin regulates TAZ protein level independent of its actin-bundling activity.

**Fig. 4:**
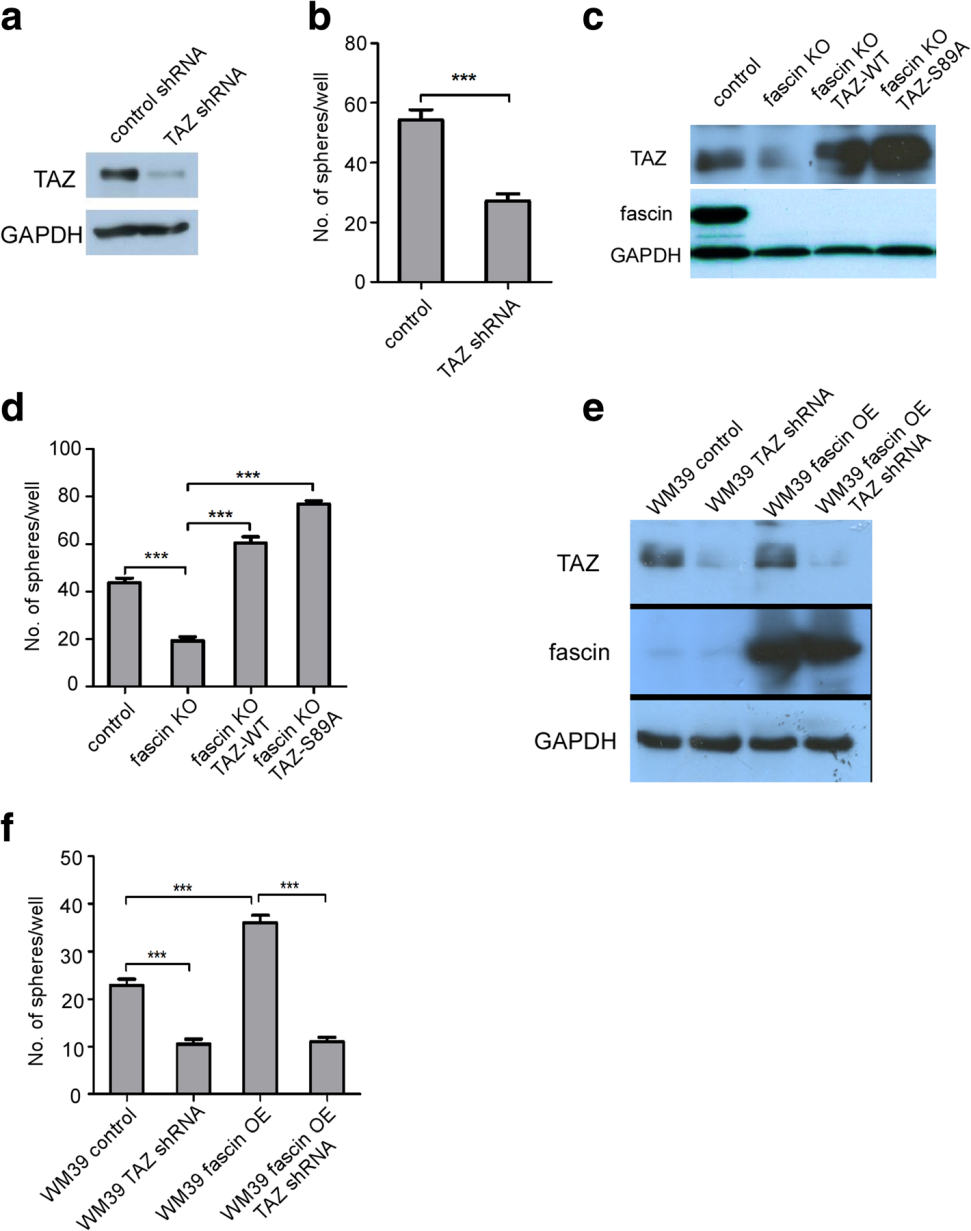
Fascin regulates melanoma sphere formation through TAZ. **a** WM793 cells were infected with control or TAZ shRNAs. Expression of TAZ was analyzed by Western blot. **b** Quantification of sphere numbers in control and TAZ knockdown WM793 cells. Data represented as mean ± SD; *n* = 3 (***P < 0.001). **c** WM793 fascin KO cells were infected with TAZ-WT and TAZ-S89A. Expression of TAZ was analyzed by Western blot. **d** Expression of TAZ-WT and TAZ-S89A overrides fascin knockdown effect on sphere growth. Data represented as mean ± SD; *n* = 3 (***P < 0.001). **e** WM39 cells were infected by TAZ shRNA and/or fascin-WT. Expression of TAZ and fascin were analyzed by Western blot. **f** Quantification of sphere numbers in control, TAZ knockdown, fascin overexpression and fascin overexpression; TAZ knockdown WM39 cells. Data represented as mean ± SD; *n* = 3 (***P < 0.001)

It has previously been shown that the level of TAZ was much higher than YAP in melanoma [16], implying that TAZ is a primary effector of the Hippo pathway in melanoma. To examine if TAZ affects melanoma cell stemness, we first knocked down TAZ using shRNA in WM793 cells (Fig. 4a) and found that depletion of TAZ decreases WM793 sphere formation by more than 50% (Fig. 4b). Based on these findings, we further investigated whether TAZ mediates fascin effect on melanoma tumorigenesis and stemness. A key regulation of TAZ is phosphorylation of TAZ at serine-89 by large tumor suppressor kinase 1 and 2 (LATS1 and LATS2). The phosphorylation of TAZ-S89 leads to its retention in cytoplasm and degradation. Mutation of TAZ-S89 to alanine, i.e., TAZ-S89A will enhance its stability and activity. Because knockout of fascin dramatically reduces melanoma stem cell growth (Fig. 1d and e) and TAZ protein level, we reasoned that expression of TAZ-wild type (WT) and TAZ-S89A should rescue fascin depletion effect on melanoma stemness. Therefore, we expressed TAZ-WT and TAZ-S89A in WM793 fascin KO cells (Fig. 4c) and found that TAZ-WT and TAZ-S89A completely abrogated fascin knockout-reduced stem cell growth (Fig. 4d). To confirm our hypothesis that fascin regulates melanoma stemness through TAZ, we employed WM39, a melanoma cell line with lower fascin expression (Additional file 1: Figure S1). Ectopic expression of fascin in WM39 cells dramatically increased sphere formation (Fig. 4e and f). Furthermore, TAZ knockdown significantly reduced stem cell growth in fascin overexpressing WM39 cells (Fig. 4e and f). These results suggested that TAZ mediates fascin function.

### Fascin regulates TAZ at protein but not mRNA level

**Fig. 5:**
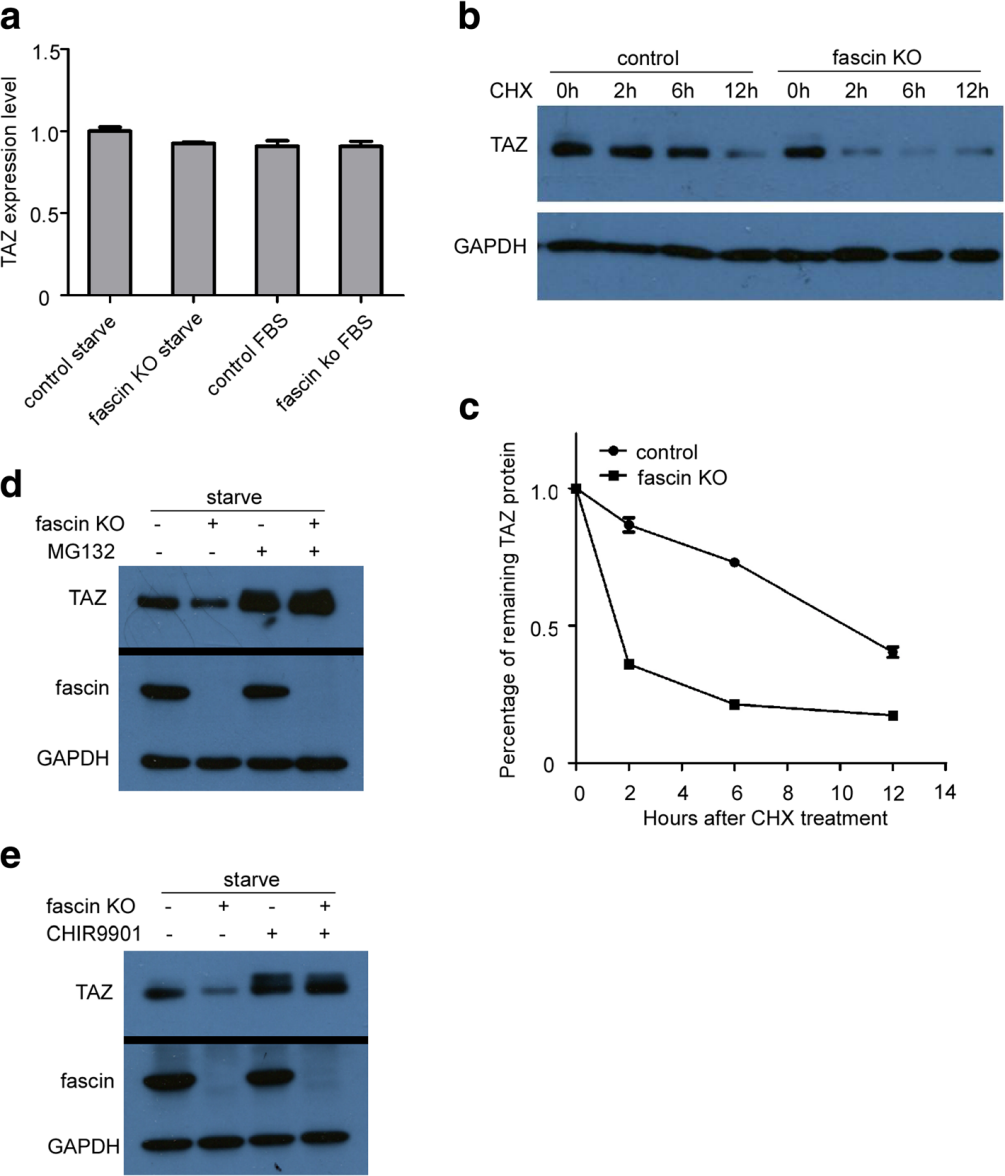
Fascin regulatesTAZ protein stability. **a** Quantitative RT-PCR analysis of TAZ mRNA level in WM793 control and WM793 fascin KO cells following culture in medium without/with serum overnight. **b** Western blot analysis of TAZ expression in WM793 control and WM793 fascin KO cells treated with cycloheximide (CHX). **c** Quantification of panel B represents TAZ degradation rate in WM793 control and WM793 fascin KO cells. **d** Immunoblotting analysis of WM793 control and WM793 fascin KO cells after serum starvation overnight and treatment with MG132 (1 μM) for indicated times. **e** Western blot analysis of WM793 control and WM793 fascin KO cells following serum starvation overnight and then treatment with GSK-3β inhibitor CHIR0091 (2 μM) for 12 h

To investigate the mechanism by which fascin affects TAZ expression, we first examined the TAZ mRNA level in WM793 control and WM793 fascin KO cells. While depletion of fascin significantly reduced TAZ protein level (Fig. 3a), we did not observe TAZ mRNA change (Fig. 5a), suggesting fascin regulation of TAZ at protein level.

We further examined whether fascin regulates TAZ protein stability. Control and fascin KO WM793 cells were treated with cycloheximide (CHX), a protein synthesis inhibitor. After 2 h of CHX treatment, TAZ level was reduced by about 20% in control WM793 cells, whereas TAZ level decreased by more than 50% in fascin KO cells. Our results indicated that TAZ degradation rate in fascin KO cells was much greater than control cells (Fig. 5b and c), suggesting that fascin regulates TAZ protein stability.

Previous study has shown that phosphorylation of TAZ-S89 leads to TAZ ubiquitination and degradation via the proteasome pathway [24]. Thus, we next tested if fascin regulates TAZ protein stability through the ubiquitin-mediated proteasome pathway. Following treatment of WM793 control and fascin KO cells with proteasome inhibitor MG132 and meanwhile starvation of the cells, we found that MG132 treatment completely inhibits TAZ degradation in fascin KO cells and restores TAZ protein expression in fascin KO cells to the same level as in control WM793 cells (Fig. 5d). These findings indicated that fascin stabilizes TAZ protein via inhibition of the ubiquitin-proteasome pathway.

In addition, recent studies showed that GSK3 phosphorylated β-catenin bridges TAZ to its ubiquitin ligase β-TrCP, leading to TAZ degradation [25, 26]. We further investigated if inhibition of the β-catenin phosphorylation rescues fascin knockout-induced TAZ degradation. Control and fascin KO WM793 cells were treated with GSK3 inhibitor CHIR0091. Immunoblotting analysis revealed that CHIR9901 treatment inhibits TAZ degradation and restores TAZ in WM793 fascin KO cell to the same level as in WM793 control cells (Fig. 5e).

### LATS1/2, upstream kinases of TAZ, are modulated by fascin

**Fig. 6:**
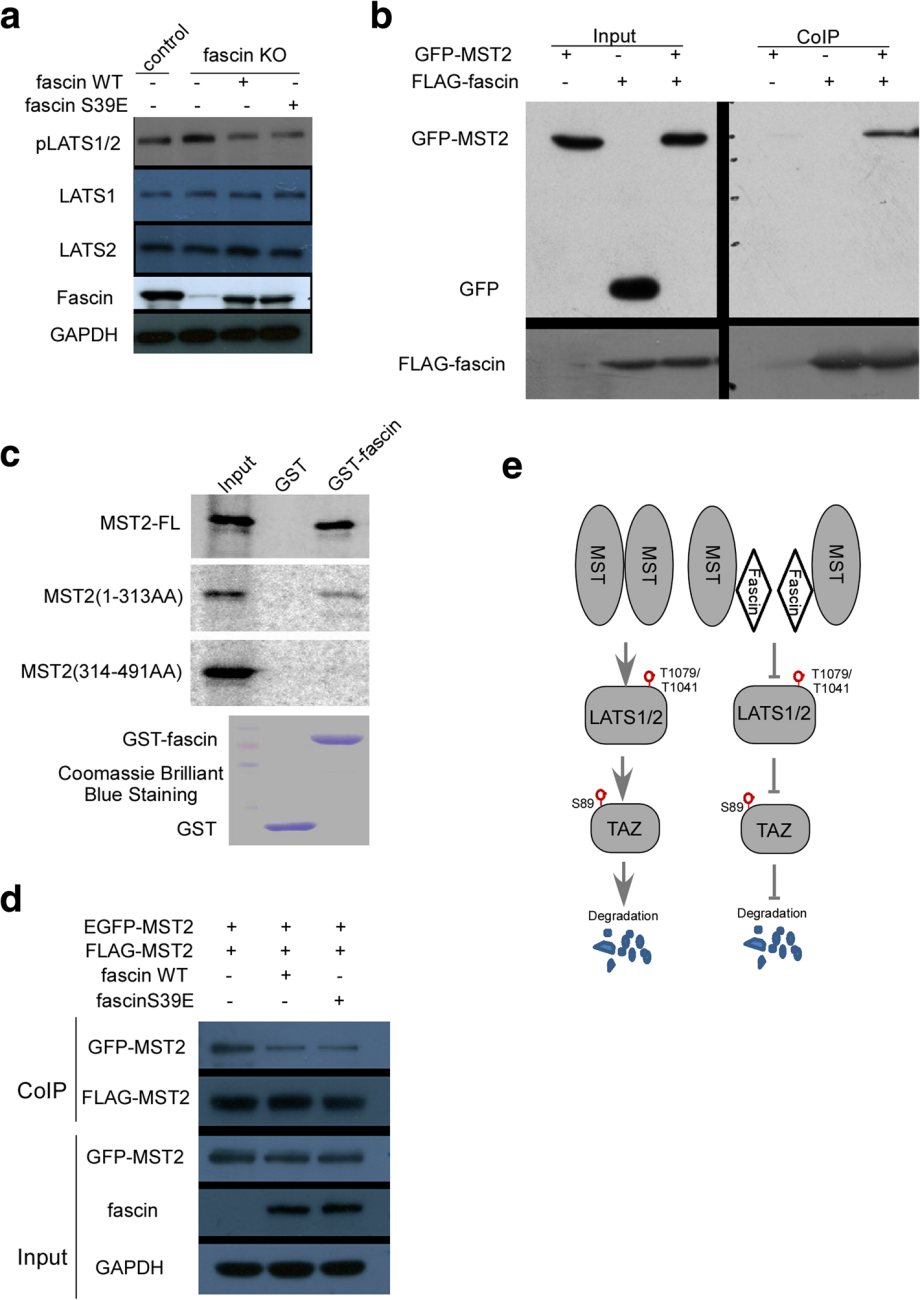
Fascin interacts with MST2 and reduces MST2 homodimeration and p-LATS. **a** WM793 fascin KO cells were reintroduced with fascin-WT and fascin-S39E plasmids. Western blot analysis was performed using indicated antibodies. **b** HEK293T cells were transfected with the indicated plasmids. Following incubation for 48 h, cell lysates were immunoprecipitated with Flag (M2) antibody and then immunoblotted with GFP antibody. **c** In vitro translated [^35^S]-MST2-full length (FL), MST2(1-313aa) and MST2(314-491aa) truncated proteins were incubated with GST and GST-fascin fusion proteins. After wash, protein complex was eluted and then separated on 10% SDS-PAGE and exposed to phospho-imager (Amersham) for autoradiography. **d** HEK293T cells were transfected with indicated plasmids. Cell lysates were immunoprecipitated with Flag (M2) antibody and then immunoblotted with GFP antibody. **e** A proposed model of TAZ regulation by Fascin. Fascin binds to MST2 and inhibits MST2 homodimer formation and kinase activity, which results in inhibition of LATS1/2 activation and TAZ phosphorylation, and thus inhibits TAZ degradation

Because TAZ is directly phosphorylated by LATS1/2 kinases and then targeted to degradation, it is logical to examine if fascin stabilizes TAZ through inhibition of LATS kinase activation. We first noticed that the levels of pLATS1/2 were dramatically elevated in WM793 fascin KO cells as compared to WM793 control cells (Fig. 6a). Reintroduction of wild-type fascin into WM793 fascin KO cells considerably reduced pLATS1/2 levels (Fig. 6a). Like fascin-S39E rescuing TAZ level, overexpression of fascin-S39E in WM793 fascin KO cells significantly reduced pLATS1/2 level (Fig. 6a).

Because LATS1/2 kinases are phosphorylated and activated by MST1/2, we hypothesized that fascin inhibition of LATS1/2 activation could be mediated by fascin interaction with MST1/2 protein. To test this, we co-transfected GFP-MST2 and Flag-fascin into HEK293T cells. Co-immunoprecipitation assay revealed that GFP-MST2 but not GFP binds to Flag-fascin (Fig. 6b). To examine if fascin directly interacts with MST2, we used in vitro GST-pulldown assay to investigate the interaction between fascin and MST2. Following incubation of GST-fascin or GST with in vitro translated MST2 and extensive washes, immunoblotting analysis showed that GST-fascin but not GST interacts with MST2. In addition, we defined the domain of MST2 that binds to fascin. MST2 protein is comprised of N-terminal kinase domain (i.e., 1–313 amino acids) and C terminal SARAH domain (i.e., 314–491 amino acids). We created MST2 truncation mutants which were used for in vitro translation. GST-pulldown assay revealed that fascin directly binds to N-terminal kinase domain but not C-terminal SARAH region of MST2 (Fig. 6c), while C-terminal SARAH domain is important for MST2 homodimer formation [27, 28]. Previous studies have demonstrated that MST2 homodimerization is required for its kinase activation [27]. Thus, we further assessed if fascin affects MST2 dimerization. HEK293T cells were co-transfected with GFP-MST2/Flag-MST2 together with wild-type fascin or loss actin-bundling activity mutant fascin-S39E. Co-immunoprecipitation showed that expression of either fascin-WT or fascin-S39E significantly reduces MST2 dimerization (Fig. 6d). Collectively, these data indicated that fascin interacts with MST2, which leads to interfering MST2 homodimer capacity and results in decreased LATS1/2 kinase activation. As a result, TAZ was stabilized by fascin inhibition of TAZ phosphorylation by LATS1/2 (Fig. 6e).

## Discussion

Melanoma is the most aggressive skin cancer and is responsible for 80% of the skin cancer-related death despite several targeted therapies, such as BRAF and MET inhibitors, have been developed. A better understanding of the pathogenesis of melanoma will lead to identify novel targets for better treatment of this disease. Fascin is upregulated in various cancers including melanoma. However, its role in melanoma and underlying mechanism are largely unknown. The significance of our present study are several folds: first demonstration of role of fascin in promoting melanoma tumorigenesis and melanoma stem cell growth; second establishment of direct link between fascin and the Hippo pathway, especially fascin regulation of TAZ but not YAP. Finally, our data indicate that fascin could be a critical therapeutic target for melanoma.

A previous study has demonstrated that fascin is frequently upregulated in melanoma, especially in metastatic lesions [29]. We have shown in the present study that knockout of fascin significantly reduces tumorigenecity and stemness of melanoma whereas ectopic expression of fascin exerts opposite effects. Because cancer stem cells are key populations of tumors for their recurrence and metastasis as well as chemoresistance, our findings indicate that targeting fascin could be an effective strategy for advanced melanoma.

Accumulating evidence suggests that the Hippo signaling pathway plays an important role in tumorigenesis and cancer stemness [13, 19]. This pathway involves a kinase cascade involving the Hippo (Hpo/MST1/2) and Warts (Wts/LATS1/2) protein kinases. Many of the genes involved in the Hippo signaling pathway are recognized as tumor suppressors, while YAP/TAZ are identified as oncogenes. YAP/TAZ can reprogram cancer cells into cancer stem cells [9, 30]. Hippo pathway is regulated by intrinsic cell machineries, such as cell–cell contact, cell polarity, and actin cytoskeleton, as well as a wide range of signals, including cellular energy status and mechanical cues. However, the role of fascin in the Hippo pathway has not been documented. We showed in this report that fascin directly interacts with MST2 and reduces MST2 homodimerization, as a result, fascin inhibits MST2 and LATS1/2 activation as well as TAZ phosphorylation and thus increases TAZ protein stability (Fig. 5e). While we did not examine if fascin binds to MST1 and inhibits MST1 kinase activity, we expect that fascin will interact with MST1 and inhibit MST1 kinase as well, because MST1 and MST2 shares high level of sequence homology especially their kinase domain.

TAZ and YAP are downstream effectors of the Hippo pathway and they share 50% identity. It has been reported that YAP and TAZ could be regulated through different mechanisms. For instance, Azzolin et al. [26] reported that TAZ, but not YAP, was regulated by Wnt. Interestingly, TAZ was expressed at higher level than YAP in melanoma, suggesting TAZ plays a more important role in melanoma tumorigenesis. A previous study showed that fascin is frequently upregulated in melanoma [29]. We presented in this study that fascin stabilizes TAZ but not YAP even though fascin inhibits MST2 and LATS1/2, the kinases upstream of both TAZ and YAP. The underlying mechanism needs further investigation. Fascin functions have been shown to primarily depend on its actin-bundling activity. However, our data show that fascin regulates melanoma tumorigenesis and the Hippo pathway independent of its actin-bundling activity (Figs. 2, 3b and 6a). Further study of the detailed mechanism is required.

Nevertheless, our study has revealed that fascin is a key regulator of the Hippo pathway. It directly binds to kinase domain of MST2 and inhibits MST/LATS kinase activity which leads to stabilization and activation of TAZ oncogenic cofactor. Furthermore, depletion of fascin dramatically reduces melanoma tumorigenesis and stem cell growth. Thus, fascin/MST/LATS/TAZ is a critical therapeutic target for melanoma.

## Conclusions

This study revealed the important role of fascin in melanoma. Fascin increases melanoma tumorigenesis and stemness via the Hippo pathway. Fascin interacts with MST2 to inhibit MST2 homodimer formation and kinase activity, and thus reduces LATS activity and enhances TAZ stability. Our study suggests that fascin/Hippo axis could be a potential therapeutic target for melanoma.

## Additional file

Additional file 1: **Figure S1.** Fascin protein levels in a panel of melanoma cell lines. Western blot analysis of fascin protein expression in 8 melanoma cell lines. (TIF 456 kb)

## Abbreviations

CHX: Cycloheximide
CRISPR: Clustered regularly interspaced short palindromic repeats
KO: Knockout
QRT-PCR: Quantitative reverse transcription polymerase chain reaction
TAZ: Transcriptional co-activator with PDZ-binding motif
YAP: Yes-Associated Protein
