# Supplementary figures and images for: Fascin induces melanoma tumorigenesis and stemness through regulating the Hippo pathway
Source: Cell Commun Signal. 2018 Jul 3;16:37. doi: 10.1186/s12964-018-0250-1 (PMC6029074; doi:10.1186/s12964-018-0250-1)

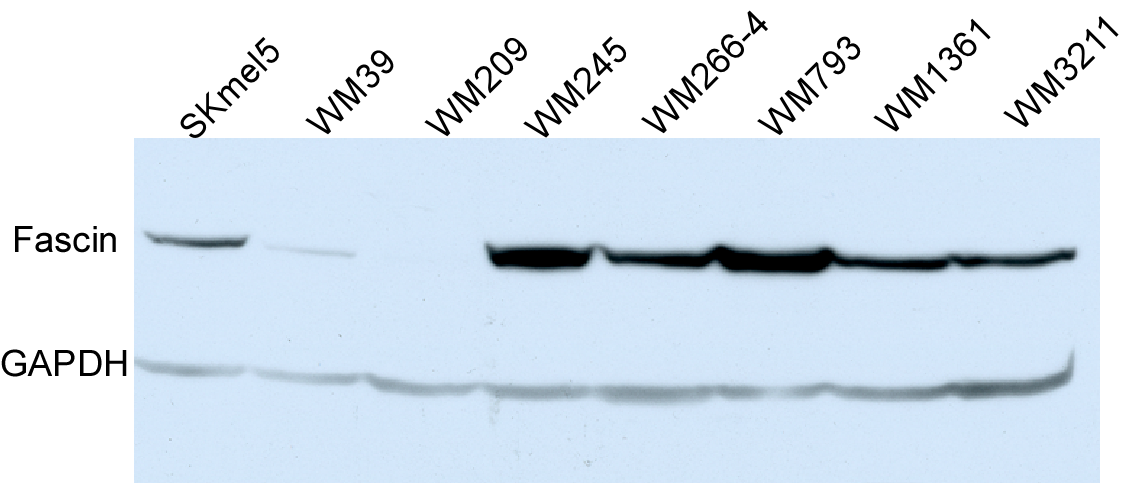

Supplement: Supplementary file 1 — Figure S1. Fascin protein levels in a panel of melanoma cell lines. Western blot analysis of fascin protein expression in 8 melanoma cell lines. (TIF 456 kb) [file 12964_2018_250_MOESM1_ESM.tif]
